# Intracorporeal and extracorporeal anastomosis for robotic-assisted and laparoscopic right colectomy: short-term outcomes of a multi-center prospective trial

**DOI:** 10.1007/s00464-021-08780-9

**Published:** 2021-11-01

**Authors:** Robert K. Cleary, Matthew Silviera, Tobi J. Reidy, James McCormick, Craig S. Johnson, Patricia Sylla, Jamie Cannon, Henry Lujan, Andrew Kassir, Ron Landmann, Wolfgang Gaertner, Edward Lee, Amir Bastawrous, Ovunc Bardakcioglu, Sushil Pandey, Vikram Attaluri, Mitchell Bernstein, Vincent Obias, Morris E. Franklin, Alessio Pigazzi

**Affiliations:** 1 Department of Surgery, Saint Joseph’s Mercy Hospital, 5325 Elliott Drive, Ste 104, Ann Arbor, MI 48106 USA; 2 Department of Surgery, Washington University School of Medicine, St. Louis, MO USA; 3 Department of Surgery, Franciscan Health, Indianapolis, IN USA; 4 Colon and Rectal Surgery, Allegheny Health Network, Pittsburgh, PA USA; 5 Department of Surgery, Oklahoma Surgical Hospital, Tulsa, OK USA; 6 Division of Colorectal Surgery, Department of Surgery, Mount Sinai Hospital, New York, NY USA; 7 Department of Surgery, University of Alabama at Birmingham, Birmingham, AL USA; 8 Colon and Rectal Surgery, Jackson Health System, Miami, FL USA; 9 Colon and Rectal Clinical, Honor Health, Scottsdale, AZ USA; 10 Department of Colon Rectal Surgery, Baptist MD Andersen Cancer Center, Jacksonville, FL USA; 11 Division of Colon and Rectal Surgery, University of Minnesota, Minneapolis, MN USA; 12 Department of Surgery, Albany Medical College, Albany, NY USA; 13 Colon and Rectal Clinic, Swedish Medical Center, Seattle, WA USA; 14 Department of Colorectal, Las Vegas School of Medicine, University of Nevada, Las Vegas, Las Vegas, NV USA; 15 West Valley Colon and Rectal Surgery Center, Sun City, AZ USA; 16 Colon and Rectal Surgery, Kaiser Permanente Los Angeles Medical Center, Los Angeles, CA USA; 17 Division of Colon and Rectal Surgery, NYU Langone Medical Center, New York, NY USA; 18 Division of Colon and Rectal Surgery, The George Washington University Hospital, Washington, DC USA; 19 Texas Endosurgery Institute, San Antonio, TX USA; 20 Division of Colon and Rectal Surgery, Weill Medical College Cornell University, New York, NY USA

**Keywords:** Robotic-assisted right colectomy, Laparoscopic right colectomy, Minimally invasive colorectal surgery, Intracorporeal anastomosis, Extracorporeal anastomosis

## Abstract

**Background:**

Studies to date show contrasting conclusions when comparing intracorporeal and extracorporeal anastomoses for minimally invasive right colectomy. Large multi-center prospective studies comparing perioperative outcomes between these two techniques are needed. The purpose of this study was to compare intracorporeal and extracorporeal anastomoses outcomes for robotic assisted and laparoscopic right colectomy.

**Methods:**

Multi-center, prospective, observational study of patients with malignant or benign disease scheduled for laparoscopic or robotic-assisted right colectomy. Outcomes included conversion rate, gastrointestinal recovery, and complication rates.

**Results:**

There were 280 patients: 156 in the robotic assisted and laparoscopic intracorporeal anastomosis (IA) group and 124 in the robotic assisted and laparoscopic extracorporeal anastomosis (EA) group. The EA group was older (mean age 67 vs*.* 65 years, *p* = 0.05) and had fewer white (81% vs. 90%, *p* = 0.05) and Hispanic (2% vs*.* 12%, *p* = 0.003) patients. The EA group had more patients with comorbidities (82% vs*.* 72%, *p* = 0.04) while there was no significant difference in individual comorbidities between groups. IA was associated with fewer conversions to open and hand-assisted laparoscopic approaches (*p* = 0.007), shorter extraction site incision length (4.9 vs*.* 6.2 cm; *p* ≤ 0.0001), and longer operative time (156.9 vs. 118.2 min). Postoperatively, patients with IA had shorter time to first flatus, (1.5 vs*.* 1.8 days; *p* ≤ 0.0001), time to first bowel movement (1.6 vs*.* 2.0 days; *p* = 0.0005), time to resume soft/regular diet (29.0 vs*.* 37.5 h; *p* = 0.0014), and shorter length of hospital stay (median, 3 vs*.* 4 days; *p* ≤ 0.0001). Postoperative complication rates were comparable between groups.

**Conclusion:**

In this prospective, multi-center study of minimally invasive right colectomy across 20 institutions, IA was associated with significant improvements in conversion rates, return of bowel function, and shorter hospital stay, as well as significantly longer operative times compared to EA. These data validate current efforts to increase training and adoption of the IA technique for minimally invasive right colectomy.

Minimally invasive options for ileocolonic anastomosis after right colectomy include extracorporeal (EA) and intracorporeal (IA) anastomotic techniques. The extracorporeal approach is characterized by minimally invasive mobilization of the diseased segment up through an extraction incision where the anastomosis is then performed by standard open methods. The extraction site for a right colectomy is typically the midline where the hernia rate is 8–12%, reportedly higher than off-midline extraction site locations. [1, 2] Mobilization of the transverse colon to reach the midline extraction site may be a technical challenge, especially in obese individuals and can result in the need to lengthen the incision. It may also result in increased bowel manipulation and mesenteric tears and bleeding, possibly contributing to increased time to gastrointestinal recovery and postoperative ileus. [1]

In contrast to the extracorporeal technique, the intracorporeal technique allows for less bowel manipulation and mobilization, improved visualization for a critical part of the operation—the anastomosis, and for the extraction site to be anywhere on the abdominal wall or through a natural orifice, such as the vagina, thereby avoiding the midline and potentially reducing the risk for incisional hernia. [2, 3] The extraction incision size is limited only by the size of the diseased segment. Furthermore, an intracorporeal anastomosis results in potential advantages, including decreased conversion to an open operation, shorter time to gastrointestinal recovery, decreased postoperative ileus, and shorter length of hospital of stay. [1, 4–7]

Previous retrospective studies comparing extracorporeal and intracorporeal techniques for right colectomy have reported inconclusive results, therefore prompting a need for a prospective analysis. The aim of this prospective multi-center observational study was to evaluate outcomes of intracorporeal and extracorporeal anastomoses using robotic assisted and laparoscopic approaches to right colectomy.

## Methods

This is a prospective, multi-center, observational study comparing intracorporeal and extracorporeal anastomoses for right colectomy. Intracorporeal and extracorporeal anastomoses were completed either via a robotic assisted or a laparoscopic approach. The study was conducted in accordance with institutional review board (IRB) guidelines and IRB approval was obtained from each participating site. Eligible patients from 20 participating institutions in the USA were recruited beginning in February 2018.

### Study design

This is an initial report of short-term outcomes up to 90 days postoperative for the ANCOR (ANastomotic COmparison in Right Colectomy) trial, a prospective study comparing IA and EA anastomoses for minimally invasive right colectomy, with specimen extraction site incisional hernia as the primary outcome.

Eligible patients were ≥ 18 years of age and scheduled to undergo either laparoscopic or robotic-assisted right colectomy for benign or malignant right colon disease (proximal to the mid transverse colon) with intracorporeal or extracorporeal anastomosis. Patients requiring emergent right colectomy and those with an obstructing, perforated, or locally invasive neoplasm (T4b), inflammatory bowel disease, or prior incisional hernia repair were excluded.

### Surgeon and operative details

Forty surgeons at 20 institutions contributed cases: 14 robotic-assisted IA surgeons, 5 laparoscopic IA surgeons, 16 laparoscopic EA surgeons, and 5 robotic-assisted EA surgeons. To ensure adequate experience, surgeons at participating sites were required to have performed a minimum of 50 right colectomies prior to contributing to a study arm. Each surgeon was limited to one surgical approach (robotic-assisted IA or robotic-assisted EA or laparoscopic IA or laparoscopic EA) and each surgeon was limited to contributing no more than 20 cases to the study.

Right colectomy for malignancy adhered to standard oncologic principles, although there were no strict criteria for the extent of mesocolic excision. All robotic-assisted procedures were performed using multi-port techniques with a da Vinci® Xi, X, or Si Surgical System.

### Data collection

Case report forms were the primary data collection instruments for this study. Each study site entered clinical data into an electronic case report form directly uploaded to a secure centralized electronic clinical database (EDC). Data entry quality was monitored by the study sponsor.

Data collected included patient demographics, operative details including operative and operating room times, conversion to open or hand-assisted laparoscopic surgery, anastomotic technique, concomitant general, colorectal, urologic, and gynecologic procedures, and postoperative outcomes, including complications, reoperation, and hospital readmission. Conversion was defined as the inability to complete an EA or IA operation without converting to open or hand-assisted laparoscopy for any reason or the need to lengthen the extraction site incision more than expected for the EA approach. The use of an enhanced recovery pathway, mechanical bowel preparation with or without antibiotics, anastomotic technique (iso- vs*.* anti-peristaltic, sutured *vs.* stapled, and anastomotic reinforcement), as well as site and length of the extraction incision were left to the discretion of the operating surgeon. Operating room time was defined as the time interval from when the patient entered the operating room to when the patient exited the operating room, and operative time was defined as time from incision to skin closure. Concomitant hepatic and other intestinal resections (in addition to right colectomy) were excluded. Ileus was defined as requiring a nasogastric tube. Data analysis was performed on an intent-to-treat basis. Consequently, conversions were analyzed under the initial operative approach, regardless of the reason for conversion.

### Statistical analysis

Standard univariate and bivariate techniques were used to describe the clinical results. Continuous variables were reported as means (and standard deviations) and median. Discrete variables (i.e., conversions, complications) were described as rates and proportions of totals. The chi-square or Fisher’s exact test was used to compare categorical or binary outcomes across groups. The independent *t* test was used for approximately normally distributed continuous outcomes, and the Wilcoxon Rank Sum test for ordinal and non-normal continuous outcomes. A *p*-value of less than 0.05 was considered statistically significant. All analyses were performed with SAS version 9.4 (SAS Institute, Inc. Cary, NC).

## Results

### Study population

**Fig. 1:**
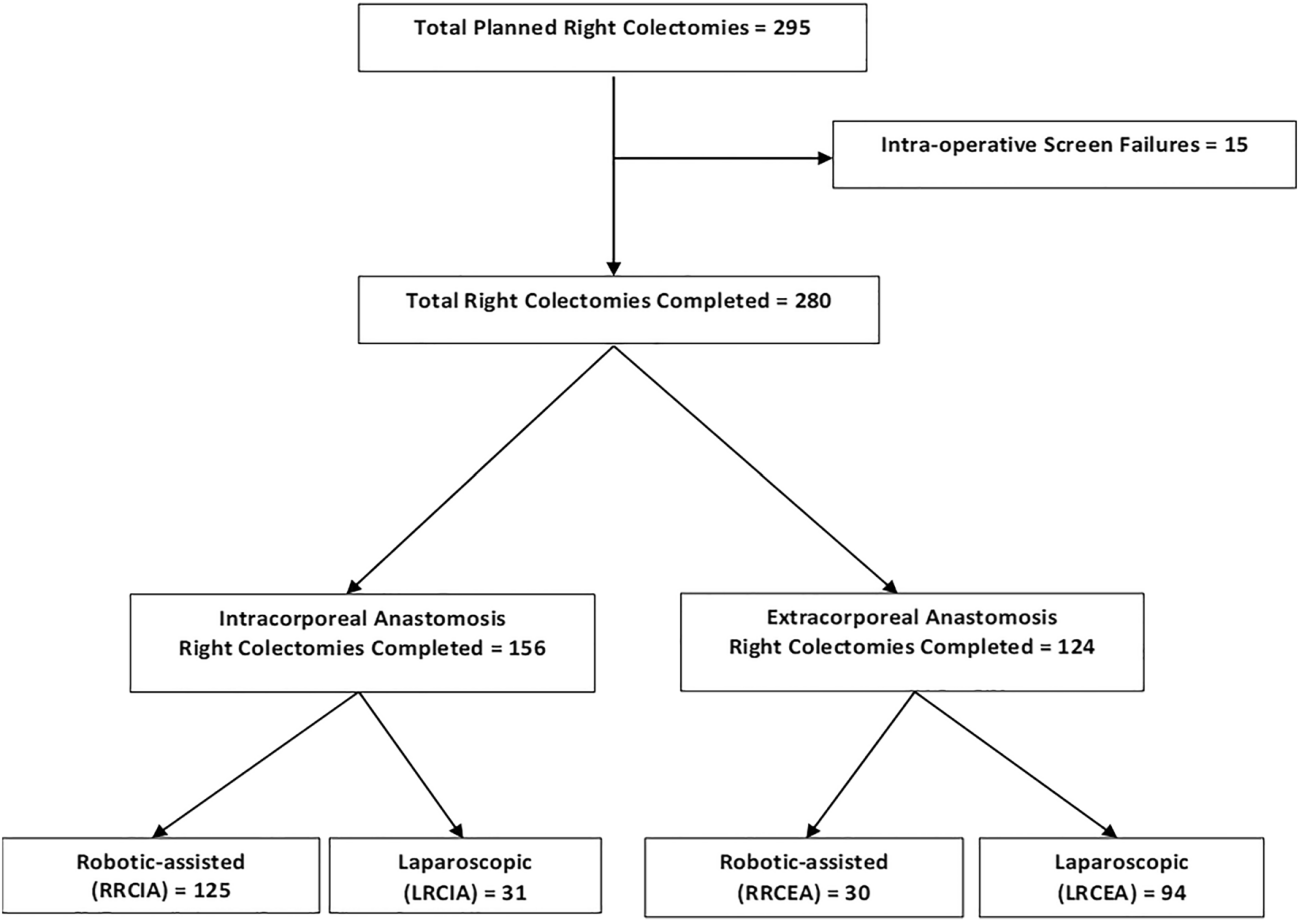
Patient distribution

Two-hundred and eighty patients met inclusion criteria and underwent minimally invasive right colectomy (Fig. 1): 156 patients underwent intracorporeal anastomosis (125 robotic assisted and 31 laparoscopic) and 124 underwent extracorporeal anastomosis (30 robotic assisted and 94 laparoscopic). Of the 156 IA cases, 90 (58%) were for malignant neoplasia and 66 (42%) were for benign neoplasia. Of the 124 EA cases, 81 (66%) were for malignant neoplasia, 42 (34%) were for benign neoplasia, and one patient had unknown tumor status. There were no significant differences between groups for operative indications (*p* = 0.104).

Sixteen surgeons performed laparoscopic EA cases, 5 performed robotic-assisted EA cases, 14 performed robotic-assisted IA cases, and 5 performed laparoscopic IA cases. Of the 16 laparoscopic EA surgeons, 12 (75%) contributed less than 10 cases and 4 (25%) contributed between 10 and 20 cases. Of the 5 robotic-assisted EA surgeons, 4 (80%) of the surgeons contributed less than 10 cases and one (20%) contributed between 10 and 20 cases. Of the 14 robotic-assisted IA surgeons, 9 (64%) of the surgeons contributed less than 10 cases and 5 (36%) contributed between 10 and 20 cases. Of the 5 laparoscopic IA surgeons, 4 (80%) of the surgeons contributed less than 10 cases and one (20%) contributed between 10 and 20 cases.

### Baseline characteristics

Table 1 shows patient characteristics for treatment groups. There were no statistically significant differences in patient demographics including sex, BMI, ASA classification, smoking status, history of abdominal or intestinal surgery, operative indication (benign or malignant neoplasia), and the use of enhanced recovery pathways. The EA group was slightly older (mean age 67 vs*.* 65 years, *p* = 0.05), with fewer white (81% vs. 90%, *p* = 0.05) and Hispanic ethnicity (2% vs. 12%, *p* = 0.003) patients. The EA group also had more patients with overall comorbidities (82% vs. 72%, *p* = 0.04), but none of the listed individual comorbidities were statistically different between groups.

**Table 1:** Patient characteristics of intracorporeal and extracorporeal groups

		IA Group (RRCIA + LRCIA) *N* = 156	EA Group (RRCEA + LRCEA) *N* = 124	*p* value
Age (years)	Mean ± SD	64.6 ± 11.1	67.2 ± 11.1	0.05
Sex, *N* (%)				0.06
	Female	73 (46.8%)	72 (58.1%)	
	Male	83 (53.2%)	52 (41.9%)	
Ethnicity, *N* (%)				0.003
	Hispanic or Latino	19 (12.2%)	3 (2.4%)	
	Not Hispanic or Latino	137 (87.8%)	121 (97.6%)	
Race, *N* (%)				0.05
	Native American	2 (1.3%)	0	
	Asian	2 (1.3%)	4 (3.2%)	
	Black	11 (7.1%)	15 (12.1%)	
	White	140 (89.7%)	100 (80.6%)	
	Other	1 (0.6%)	5 (4.0%)	
BMI	Mean ± SD	30.4 ± 7.2	29.6 ± 5.6	0.30
ASA Classification, *N* (%)				0.33
	ASA Class 1	7 (4.5%)	4 (3.2%)	
	ASA Class 2	58 (37.2%)	46 (37.1%)	
	ASA Class 3	85 (54.5%)	60 (48.4%)	
	ASA Class 4	6 (3.8%)	14 (11.3%)	
≥ 1 comorbidities, *N* (%)		112 (71.8%)	102 (82.3%)	0.04
	Hypertension	86 (55.1%)	79 (63.7%)	–
	Myocardial infarction	10 (6.4%)	6 (4.8%)	–
	Congestive heart failure	4 (2.6%)	9 (7.3%)	–
	Coronary artery disease	20 (12.8%)	20 (16.1%)	–
	Peripheral vascular disease	8 (5.1%)	7 (5.6%)	–
	Cerebrovascular disease	10 (6.4%)	6 (4.8%)	–
	COPD	8 (5.1%)	7 (5.6%)	–
	Diabetes	29 (18.5%)	22 (17.7%)	–
	Moderate/severe renal disease	4 (2.6%)	9 (7.3%)	–
	Chronic steroid immunosuppressive use	2 (1.3%)	2 (1.6%)	–
Smoking status, *N* (%)				0.9373
	Never smoked	100 (64.1%)	82 (66.1%)	
	Past smoker	43 (27.6%)	32 (25.8%)	
	Current smoker	13 (8.3%)	10 (8.1%)	
Previous intestinal surgery, *N* (%)		54 (34.6%)	36 (29.0%)	0.32
Indication for surgery, *N* (%)				0.10
	Benign neoplasm	76 (48.7%)	46 (37.4%)	
	Malignant neoplasm	79 (50.6%)	76 (61.8%)	
	Other	1 (0.6%)	1 (0.8%)	
ERP, *N* (%)		100 (64.1%)	81 (65.3%)	0.761

*RRCIA* robotic-assisted right colectomy intracorporeal anastomosis, *LRCIA* laparoscopic right colectomy intracorporeal anastomosis, *RRCEA* robotic-assisted right colectomy extracorporeal anastomosis, *LRCEA* laparoscopic right colectomy extracorporeal anastomosis, *BMI* body mass index, *ASA* American Society of Anesthesiologists, *SD* standard deviation of the mean, *COPD* chronic obstructive pulmonary disease, *ERP* enhanced recovery pathway

### Operative outcomes

Intracorporeal anastomosis was associated with significantly longer mean operating room (208.5 vs. 175.5 min, *p* < 0.0001) and mean operative times (157 vs. 118 min, *p* < 0.0001 [Table 2]). Conversion was significantly lower in IA patients compared to the EA group (0% vs. 5%, *p* = 0.007). Of the 6 extracorporeal conversions, 5 were to open and 1 to hand-assisted laparoscopy. The reasons for conversion were abdominal adhesions (*n* = 4) and morbid obesity (*n* = 2). Most of the extracorporeal anastomoses were anti-peristaltic (87%) while most of the intracorporeal anastomoses were iso-peristaltic (95.5%).

**Table 2:** Operative outcomes

		IA Group (RRCIA + LRCIA) *N* = 156	EA Group (RRCEA + LRCEA) *N* = 124	*p* value
OR time (min) (Wheels-in to Wheels-out)	Mean ± SD	208.5 ± 55.9	175.5 (56.0), 124	< 0.0001
Operative time (min) (Skin-to-skin)	Mean ± SD	156.9 ± 50.2	118.2 ± 43.5	< 0.0001
Conversion, *N* (%)		0	6 (4.8%	0.007
	To open	0	5 (4.0%)	
	To hand-assisted lap	0	1 (0.8%)	
Anastomosis, *N* (%)	Iso-peristaltic	149 (95.5%)	15 (12.1%)	< 0.0001
	Anti-peristaltic	7 (4.5%)	108 (87.1%)	
Specimen Extraction, *N* (%)	Midline	2 (1.3%)	123 (100%)	< 0.0001
	*Off-Midline:*	154 (98.7%)	0	
	*Pfannenstiel*	121 (77.6%)	0	–
	*McBurney's*	1 (0.6%)	0	–
	*Paramedian*	7 (4.5%)	0	–
	*Other*	25 (16.0%)	0	–
	*Unknown*	0	1	–
Concomitant procedures, *N* (%)		6 (3.8%)	15 (12.1%)	0.009
	General surgery	6 (3.8%)	14 (11.3%)	
	Colorectal	0	1 (0.8%)	
Extraction Incision Length (cm)	Mean ± SD [*n*]	4.9 ± 1.4	6.2 ± 3.6 [123]	< 0.0001
	Intraoperative blood transfusion, *N* (%)	2 (1.3%)	1 (0.8%)	> 0.99
	Intraoperative complications, *N* (%)	1 (0.6%)	0	–

RRCIA = robotic-assisted right colectomy intracorporeal anastomosis, LRCIA = laparoscopic right colectomy intracorporeal anastomosis, RRCEA = robotic-assisted right colectomy extracorporeal anastomosis, LRCEA = laparoscopic right colectomy extracorporeal anastomosis

SD = standard deviation of the mean, OR = operating room, PACU = post-anesthesia care unit

The majority of extracorporeal specimen extraction incisions were at the midline (100%), while 99% of the intracorporeal specimen extraction incisions were located off-midline (Pfannenstiel 78%, paramedian 4.5%, other 16%, and McBurney’s point 0.6%). Patients in EA group had more concomitant procedures than patients in the IA group [12% vs. 4%, *p* = 0.009 (Table 2)].

The mean extraction site incision length was significantly longer in the EA group (6.2 cm vs. 4.9 cm, *p* ≤ 0.0001) compared to the IA group. Two patients in IA group and one patient in EA group received intraoperative blood transfusions. Only one patient in the IA group experienced an intraoperative complication, a bladder injury that occurred while making a Pfannenstiel extraction site incision. This injury was recognized immediately and easily repaired.

### Postoperative outcomes

Table 3 shows postoperative outcomes prior to discharge. Time to first flatus (1.5 vs. 1.8 days, *p* < 0.0001), time to first bowel movement (1.6 vs*.* 2.0 days, *p* = 0.0005), and time to soft/regular diet (1.2 vs*.* 1.6 days, *p* = 0.0014) were all significantly shorter in the IA group. Length of hospital stay was significantly shorter in the IA group (median, 3 vs*.* 4 days, *p* < 0.0001). There was no significant difference between groups in discharge to home (IA 98.1% vs. EA 96.0%) or discharge to an extended care facility (IA 1.9% vs. 0.8%). There was 1 death in the EA group.

**Table 3:** Postoperative outcomes

	IA Group (RRCIA + LRCIA) *N* = 156	EA Group (RRCEA + LRCEA) *N* = 124	*p* value
Days to first flatus			
Mean ± SD [*n*]	1.5 ± 1.0 [152]	1.8 ± 1.0 [121]	< 0.0001
Days to first bowel movement			
Mean ± SD [*n*]	1.6 ± 0.9 [153]	2.0 ± 1.1 [118]	0.0005
Days to soft/regular diet			
Mean ± SD [*n*]	1.2 ± 25.2	1.6 ± 27.9 [123]	0.0014
Hospital LOS (days)			
Mean ± SD [*n*]	4.2 ± 3.1	4.4 ± 1.5 [122]	< 0.0001
Median (IQR)	3.0 (3.0, 4.0)	4.0 (3.0, 5.0)	
Discharge status, *N* (%)			
Home	153 (98.1%)	119 (96.0%)	0.46
Care facility	3 (1.9%)	1 (0.8%)	
Death prior to discharge	0	1.0 (0.8%)	

*RRCIA* robotic-assisted right colectomy intracorporeal anastomosis, *LRCIA* laparoscopic right colectomy intracorporeal anastomosis, *RRCEA* robotic-assisted right colectomy extracorporeal anastomosis, *LRCEA* laparoscopic right colectomy extracorporeal anastomosis, *SD* standard deviation of the mean

Table 4 shows postoperative complications. There were no significant differences in overall postoperative complications prior to discharge between groups (IA 10% vs*.* EA 8%, *p* = 0.65). This lack of significant difference between groups persisted at 14 days (IA 3% vs*.* EA 2%, *p* = 0.99) and 90 days (IA 1% vs*.* EA 0%) after discharge. Anastomotic leaks (IA 0.6% vs. EA 0%), surgical site infections (1.3% vs. 0%), hospital readmission (IA 2.6% vs. EA 0.8%, *p* = 0.387), and reoperations (0.6% vs. 0%, *p* > 0.99) were also comparable between groups.

**Table 4:** Postoperative complications

	IA Group (RRCIA + LRCIA) *N* = 156	EA Group (RRCEA + LRCEA) *N* = 124	*p* value
Postoperative complications to discharge^a^, *N* (%)	15 (9.6%)	10 (8.1%)	0.6512
Gastrointestinal	8 (5.1%)	3 (2.4%)	–
Ileus	7 (4.5%)	2 (1.6%)	–
Anastomotic leakage	1 (0.6%)	0	–
Bowel obstruction	0	1 (0.8%)	–
Bleeding requiring intervention	3 (1.9%)	2 (1.6%)	–
Wound	2 (1.3%)	1 (0.8%)	–
Superficial SSI	2 (1.3%)	0	–
Wound dehiscence^b^	0	1 (0.8%)	–
Cardiac	1 (0.6%)	1 (0.8%)	–
Pulmonary	1 (0.6%)	1 (0.8%)	–
Genitourinary	3 (1.9%)	4 (3.2%)	–
Complications^a^: discharge to 2 weeks, *N* (%)	5 (3.2%)	3 (2.4%)	> 0.99
Gastrointestinal	2 (1.3%)	1 (0.8%)	–
Deep SSI	0	1 (0.8%)	–
Wound	1 (0.6%)	0	–
Genitourinary	1 (0.6%)	1 (0.8%)	–
Readmissions, *N* (%)	4 (2.6%)	1 (0.8%)	0.3869
Reoperations, *N* (%)	1 (0.6%)	0 (0.0%)	> 0.99

*RRCIA* robotic-assisted right colectomy intracorporeal anastomosis, *LRCIA* laparoscopic right colectomy intracorporeal anastomosis, *RRCEA* robotic-assisted right colectomy extracorporeal anastomosis, *LRCEA* laparoscopic right colectomy extracorporeal anastomosis, *SSI* surgical site infection

^a^Complications requiring invasive intervention

^b^At specimen extraction site

Short-term oncologic outcomes are presented in Table 5. Mean tumor size for malignant cases was 3.7 cm in the IA group and 4.2 cm in the EA group (*p* = 0.225). There were no significant differences in tumor location or TNM staging between groups. Mean lymph node harvest was 23 in the IA group and 24 in the EA group (*p* = 0.535), with no significant differences in mean number of positive lymph nodes (1.4 vs*.* 1.6, *p* = 0.403), respectively. Of those with malignant disease who received adjuvant chemotherapy (IA 29% vs. EA 33%, *p* = 0.605), there were no significant delays in starting treatment, with a mean time from surgery to chemotherapy of 40 days (IA group) versus 46 days (EA group) (*p* = 0.277).

**Table 5:** Pathologic and adjuvant therapy outcomes for malignant neoplasia cases

	IA Group (RRCIA + LRCIA) *N* = 90	EA Group (RRCEA + LRCEA) *N* = 81	*p* value
Tumor size (cm)			
Mean ± SD [*n*]	3.7 ± 2.3 [89]	4.2 ± 2.5 [80]	0.225
TNM stage, *N* (%)			
Stage 0	0	4 (4.9%)	0.8004
Stage I	28 (31.5%)	22 (27.2%)	
Stage II	19 (12.2%)	18 (14.5%)	
Stage III	39 (43.8%)	33 (40.7%)	
Stage IV	3 (3.3%)	4 (4.9%)	
Tumor Location, *N* (%)			
Cecum	47 (52.2%)	34 (42.0%)	0.1801
Ascending colon	30 (33.3%)	38 (46.9%)	
Hepatic flexure	10 (11.1%)	5 (6.2%)	
Transverse colon	2 (2.2%)	4 (4.9%)	
Terminal Ileum	1 (1.1%)	0	
Lymph node harvest			
Mean ± SD [*n*]	23.3 ± 10.0 [89]	24.2 ± 9.5 [81]	0.535
Number lymph nodes positive			
Mean ± SD [*n*]	1.4 ± 2.7 [89]	1.6 ± 5.5 [81]	0.403
Adjuvant chemotherapy, *N* (%)	26 (29.2%)	26 (32.9%)	0.605
Time to chemotherapy (days)			
Mean ± SD [*n*]	39.8 ± 14.5 [26]	46.0 ± 20.2 [26]	0.277

*RRCIA* robotic-assisted right colectomy intracorporeal anastomosis, *LRCIA* laparoscopic right colectomy intracorporeal anastomosis, *RRCEA* robotic-assisted right colectomy extracorporeal anastomosis, *LRCEA* laparoscopic right colectomy extracorporeal anastomosis

## Discussion

This prospective, multi-center, comparative study across 20 institutions comparing intracorporeal and extracorporeal anastomoses for robotic assisted and laparoscopic right colectomy for benign and malignant disease demonstrated significant advantages with the intracorporeal approach showing fewer conversions to open surgery, shorter extraction site incision, shorter time to gastrointestinal recovery, and shorter length of hospital stay. The IA technique was associated with longer operative times when compared to the EA approach. Postoperative complications were comparable between the two groups.

Previous studies have confirmed advantages with IA. Four meta-analyses have shown shorter time to return of bowel function, shorter length of hospital stay, and less postoperative morbidity with IA when compared to EA [6, 8–10]. Although the mechanism by which bowel function recovers faster in IA patients is unknown, hypotheses include less bowel manipulation and dissection and a predominance of iso-peristaltic anastomoses with the intracorporeal technique. Length of hospital stay is a parameter that is influenced by patient and non-patient factors and the use of enhanced recovery pathways. Recovery of bowel function has been reported to be shorter for IA in retrospective studies, although other smaller, retrospective, single-institution studies have also shown no difference when compared to EA [6, 8, 11–13]. In the present study, there was no significant difference in the use of enhanced recovery pathways (IA 64% vs*.* EA 65%, *p* = 0.761).

A randomized controlled trial of 140 patients comparing laparoscopic IA and EA found that operative time was significantly longer in the IA group and that time to gastrointestinal recovery, ileus, and postoperative complications were significantly less in the IA group [11]. In contrast to our study, the number of patients in this randomized trial was smaller and all patients underwent a laparoscopic approach. The primary outcome was length of hospital stay, which was longer than typically expected for minimally invasive right colectomy (IA 5.7 days vs*.* EA 6.6 days, *p* = 0.194). The incision length for both groups was also unusually long and significantly different (IA 6.7 cm vs*.* EA 8.7 cm, *p* < 0.001). Incision length in our study was also significantly different between groups in favor of the IA technique (IA 4.9 cm vs*.* EA 6.2 cm, *p* < 0.0001).

Other studies have also suggested that IA is associated with fewer complications than EA [14, 15]. In a retrospective propensity score-matched analysis of 1029 patients, IA showed advantages in conversion, length of hospital stay, and postoperative complications [16]. It is possible that the IA technique, especially with the laparoscopic approach, requires a skill set that decreases the risk for conversion during the colon and mesentery mobilization parts of the procedure. Also, EA conversion may occur when extension of the extraction site incision is necessary to enable transverse colon reach, an operative step that is not part of the IA technique. Our study showed a significantly shorter length of hospital stay for the IA group that was not related to the incidence of ileus. Although not statistically significant, the incidence of ileus was higher in the IA than in the EA group (4.5% vs. 1.6%). Differences in institutional ERP methods and discharge criteria can impact length of hospital stay. ERP was included in the statistical model but standardized discharge criteria were not and this may be considered a study limitation. Our current prospective study did not confirm an advantage of IA in postoperative complications, although the overall number of complications was low. Comparable to the larger retrospective study mentioned above, operative times for IA were longer compared to EA.

Laparoscopic IA is not a common minimally invasive operative approach choice given the skills required to accomplish this technique. The robotic approach has increased the adoption of IA due to the benefits of endowrist articulated instruments that permit precise dissection, suturing, and stapling with seven degrees of freedom, allowing IA to be amenable to more surgeon skill sets than the laparoscopic counterpart. The degree of difficulty of the sutured laparoscopic anastomosis has limited the widespread adoption of this approach and may be the reason for lower IA technique study numbers in many studies, as was the case in ours [12]. In a randomized clinical trial by Park et al*.*, [17] comparing the short-term outcomes of robotic assisted *versus* standard laparoscopic right colectomy, IA was performed more often with the robotic-assisted approach, whereas EA was more often performed with the laparoscopic technique. Our study design took into account the anticipated difficulties recruiting laparoscopic surgeons accruing laparoscopic IA cases.

The strength of this multi-center comparative study is that the results may be generalizable and representative of the real-world setting. It validates prior single-institution studies published by MIS experts. There are some limitations. Patients with ileocecal Crohn’s disease benefit from a minimally invasive approach but outcomes for these patients with nutritional deficits and on immunosuppressive medications may be different than for benign and malignant neoplasia. We chose to concentrate on a relatively uniform patient population to compare IA and EA and therefore excluded patients with Crohn’s disease. We could not control for preoperative interventions, such as mechanical bowel preparation and the specific elements of enhanced recovery pathways, and there was no unified method for diet resumption across all centers. We reviewed the significant differences in patient demographics shown in Table 1 and these may be attributed to regional population distribution differences that were unlikely to contribute significantly to clinical outcomes alone. The study design did not account for racial differences. This study involved an uncommonly higher number of institutions and surgeons, which may have contributed to increased variability and data heterogeneity, although this was necessary to accrue the number of patients for each group in a reasonable amount of time. Although experienced minimally invasive surgeons were instructed to adhere to IA and EA principles, they were limited to one technique and 20 cases total to allow homogeneous and balanced case contributions per surgeon and institution. We did not choose a randomized controlled design so that surgeons would not perform operations uncommon to their practice, such as laparoscopic IA. Also, we could not control for the degree of intracorporeal mobilization prior to extracorporeal extraction.

This study demonstrates significant advantages for the IA compared to the EA technique, whether the approach is laparoscopic or robotic. These data validate the value of minimally invasive right colectomy and the benefits of the IA technique. Further studies comparing laparoscopic *versus* robotic-assisted IA may be warranted and should focus on operative proficiency and the benefits of iso- *versus* anti-peristaltic anastomotic orientation.

## Conclusion

In this prospective multi-center study of minimally invasive right colectomy across 20 institutions, IA was associated with significant improvements in short-term outcomes including conversion to open surgery, quicker return of bowel function, and shorter length of hospital stay. Operative times were significantly longer in the IA group. These outcome advantages support current and future training programs preparing surgeons in the adoption of intracorporeal minimally invasive surgery techniques.

## References

[1] Morpurgo E, Contardo T, Molaro R, Zerbinati A, Orsini C, D'Annibale A (2013). Robotic-assisted intracorporeal anastomosis versus extracorporeal anastomosis in laparoscopic right hemicolectomy for cancer: a case control study. J Laparoendosc Adv Surg Techn Part A.

[2] Samia H, Lawrence J, Nobel T, Stein S, Champagne BJ, Delaney CP (2013) Extraction site location and incisional hernias after laparoscopic colorectal surgery: should we be avoiding the midline? Am J Surg. 205(3):264–7. (**discussion 8**) 10.1016/j.amjsurg.2013.01.006 23375702

[3] Harr JN, Juo Y-Y, Luka S, Agarwal S, Brody F, Obias V (2016). Incisional and port-site hernias following robotic colorectal surgery. Surg Endosc.

[4] Trastulli S, Coratti A, Guarino S, Piagnerelli R, Annecchiarico M, Coratti F (2014). Robotic right colectomy with intracorporeal anastomosis compared with laparoscopic right colectomy with extracorporeal and intracorporeal anastomosis: a retrospective multicentre study. Surg Endosc.

[5] Cirocchi R, Trastulli S, Farinella E, Guarino S, Desiderio J, Boselli C (2013). Intracorporeal versus extracorporeal anastomosis during laparoscopic right hemicolectomy: systematic review and meta-analysis. Surg Oncol.

[6] Feroci F, Lenzi E, Garzi A, Vannucchi A, Cantafio S, Scatizzi M (2013). Intracorporeal versus extracorporeal anastomosis after laparoscopic right hemicolectomy for cancer: a systematic review and meta-analysis. Int J Colorectal Dis.

[7] Tarta C, Bishawi M, Bergamaschi R (2013). Intracorporeal ileocolic anastomosis: a review. Tech Coloproctol.

[8] Ricci C, Casadei R, Alagna V, Zani E, Taffurelli G, Pacilio CA (2017). A critical and comprehensive systematic review and meta-analysis of studies comparing intracorporeal and extracorporeal anastomosis in laparoscopic right hemicolectomy. Langenbeck Arch Surg.

[9] van Oostendorp S, Elfrink A, Borstlap W, Schoonmade L, Sietses C, Meijerink J (2017). Intracorporeal versus extracorporeal anastomosis in right hemicolectomy: a systematic review and meta-analysis. Surg Endosc.

[10] Milone M, Elmore U, Vignali A, Gennarelli N, Manigrasso M, Burati M (2018). Recovery after intracorporeal anastomosis in laparoscopic right hemicolectomy: a systematic review and meta-analysis. Langenbeck's Arch Surg.

[11] Bollo J, Turrado V, Rabal A, Carrillo E, Gich I, Martinez MC (2020). Randomized clinical trial of intracorporeal versus extracorporeal anastomosis in laparoscopic right colectomy (IEA trial). Br J Surg.

[12] Lujan HJ, Plasencia G, Rivera BX, Molano A, Fagenson A, Jane LA (2018). Advantages of robotic right colectomy with intracorporeal anastomosis. Surg Laparosc Endosc Percutaneous Techn.

[13] Hanna MH, Hwang GS, Phelan MJ, Bui T-L, Carmichael JC, Mills SD (2016). Laparoscopic right hemicolectomy: short- and long-term outcomes of intracorporeal versus extracorporeal anastomosis. Surg Endosc.

[14] Akram W, Al-Natour R, Albright J, Wu J, Ferraro J, Shanker B (2018). A propensity score-matched comparison of intracorporeal and extracorporeal techniques for robotic-assisted right colectomy in an enhanced recovery pathway. Am J Surg.

[15] Shapiro R, Keler U, Segev L, Sarna S, Hatib K, Hazzan D (2016) Laparoscopic right hemicolectomy with intracorporeal anastomosis: short- and long-term benefits in comparison with extracorporeal anastomosis. Surg Endosc 30(9):3823–3829 10.1007/s00464-015-4684-x 26659237

[16] Cleary RK, Kassir A, Johnson CS, Bastawrous AL, Soliman MK, Marx DS (2018). Intracorporeal versus extracorporeal anastomosis for minimally invasive right colectomy: a multi-center propensity score-matched comparison of outcomes. PLoS ONE.

[17] Park JSC, Park SY, Kim HJ, Ryuk JP (2012) Randomized clinical trial of robot-assisted versus standard laparoscopic right colectomy. Br J Surg 99(9):1219–1226 10.1002/bjs.8841 22864881

